# Anticancer activity of RM-3-22: a TAZQ-based hydroxamic acid derivative targeting NSCLC in vitro and in vivo

**DOI:** 10.3389/fphar.2025.1544666

**Published:** 2025-06-05

**Authors:** Essha Chatterjee, Ram Sharma, Biswajit Dey, Hoshiyar Singh, Aliva Naik, Anjesh Khan, Santanu Basak, Ankush Bansode, Ritika Sachdeva, Anamika Sharma, Rahul Kumar, Pradeep Naik, Pankaj Kumar Singh, Kunal Nepali, Santosh Kumar Guru

**Affiliations:** ^1^ Department of Biological Sciences, National Institute of Pharmaceutical Education and Research, Hyderabad, India; ^2^ Department of Biotechnology and Bioinformatics, Sambalpur University, Sambalpur, Odisha, India; ^3^ School of Pharmacy, College of Pharmacy, Taipei Medical University, Taipei, Taiwan; ^4^ Department of Pharmaceutics, National Institute of Pharmaceutical Education and Research, Hyderabad, India

**Keywords:** apoptosis, autophagy, cell cycle arrest, cell death, PI3K/Akt/mTOR pathway

## Abstract

**Introduction:**

Lung cancer remains the leading cause of cancer-related deaths, necessitating novel therapeutic strategies. In this study, we developed RM-3-22, a TAZQ-based hydroxamic acid derivative with histone deacetylase (HDAC) inhibitory properties. We evaluated its anticancer activity in non-small cell lung cancer (NSCLC), using A549 adenocarcinoma cells as the primary model.

**Methods:**

The anticancer efficiency of RM-3-22 was assessed in 2D and 3D cell culture models. Cell survivalism was analysed by MTT assay. Different microscopical staining methods, including acridine orange and DAPI, were employed to evaluate autophagy, nuclear changes, and apoptosis. Cell cycle progression, mitochondrial membrane potential, and apoptosis-necrosis profiles were assessed using flow cytometry. Protein and gene expression related to the RM-3-22 induced pathway were evaluated via immunofluorescence (IF), Western blotting, and RT-PCR. Functional gene analysis was performed using siRNA-mediated knockdown. Different in silico studies were also conducted to check the clinical relevance and expression pattern of the RM-3-22-induced gene. Additionally, the in vivo efficiency of the molecule was evaluated using the NOD/SCID xenograft model.

**Results:**

RM-3-22 potentially suppressed cell viability and decreased the tumor spheroid size of A549s in vitro. It induced autophagy via downregulation of PI3K/Akt/mTOR signalling pathway. Besides, flow cytometry confirmed increased apoptotic cell population and decreased mitochondrial membrane potential due to the exposure of RM-3-22. RM-3-22 also promoted G2/M arrest. Signalling cascade confirmed that autophagy regulates RM-3-22-mediated apoptosis and cell cycle arrest. Additionally, RM-3-22 upregulated FTH1, a tumor suppressor, reinforcing its anticancer potential. Notably, RM-3-22 exhibited lower toxicity to normal cells, underscoring its selectivity. In vivo, RM-3-22 markedly reduced tumor growth in the xenograft mouse model.

**Conclusion:**

RM-3-22 demonstrates potent anticancer activity through different mechanisms, including inhibition of the PI3K/Akt/mTOR pathway and activation of autophagy, apoptosis, and cell cycle arrest. Further, in vivo validation also supports that RM-3-22 represents a promising therapeutic candidate against lung cancer.

## Graphical Abstract

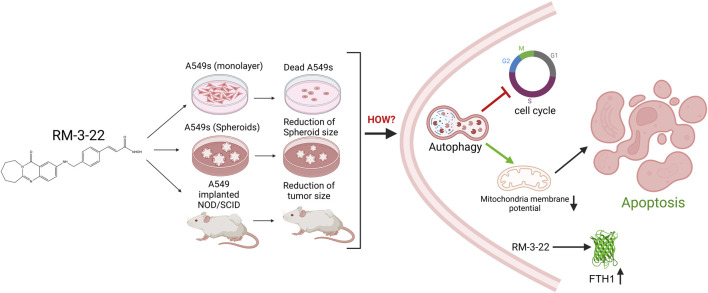

## Highlights

1. RM-3-22 is a potent anticancer molecule, specifically for lung cancer.
2. It induces autophagy and the intrinsic form of apoptosis.
3. It causes cell cycle arrest at the G2/M phase.
4. The signaling cascade that leads to the death of lung cancer cell lines caused by RM-3-22 is furnished.
5. It induces FTH1 expression. FTH1 acts as a tumor suppressor gene in lung cancer.

## 1 Introduction

Lung cancer is the most prevalent cancer type among cancer-related deaths worldwide (Wheless et al., 2013; de Groot et al., 2018; Sung et al., 2021). Lung cancer can be classified into non-small-cell carcinoma (NSCLC) and small-cell carcinoma (SCLC). Specifically, NSCLC accounts for approximately 85% of all lung cancer cases (Molina et al., 2008; Terrones et al., 2023). So, new pharmaceutical entities are needed to cure this disease.

In contemporary studies, histone deacetylase inhibitors (HDACis) are among the most widely studied compounds because of their anticancer activities (Eckschlager et al., 2017; Li and Seto, 2016).

HDAC inhibitors can reverse epigenetic modifications, leading to the suppression of oncogenic pathways and reactivation of tumor suppressor genes. Inducing the histone acetylation, they generate a more relaxed chromatin structure that facilitates the transcription of genes involved in the cell cycle regulation and cell death pathway (Li et al., 2020). Moreover, in combination therapy with existing cancer treatments, HDACis have been shown to induce the efficacy of the treatment outcome (Hontecillas-Prieto et al., 2020).

HDACis could be exemplified by trichostatin A (TSA), panobinostat (Farydak), suberoylanilide hydroxamic acid (SAHA), etc. (Bondarev et al., 2021). Interestingly, SAHA is the first pan-histone deacetylase inhibitor approved by the Food and Drug Administration (FDA) to treat cutaneous T-cell lymphoma (CTCL) (Wawruszak et al., 2021; Huang et al., 2020). Other HDACis have also shown efficacy in hematological cancers but have shown limited success in treating solid tumors, such as breast and lung cancers (Peela et al., 2017; Zhao et al., 2014). In addition, HDACis are associated with several side effects (Shi et al., 2024). Relevant toxicity was observed in the SAHA-treated mouse model in high-dose exposure, and it also exhibited several side effects in patients during its clinical trial (Nervi et al., 2015). To solve this, scientists experimented with low doses of SAHA in combination with other drugs, such as 5-fluorouracil (5-FU) and paclitaxel, to treat several cancers (Noro et al., 2010; Chao et al., 2015; Cooper et al., 2007). However, combinational therapy in cancer is always challenging because of the narrow therapeutic index of each drug owing to overlapping toxicities and difficulty in determining the best combination of two drugs and combining those in a clinical setting (Lopez and Banerji, 2017).

In this context, we previously developed a synthetic HDACi, tetrahydroazepino quinazolinone (TAZQ)-based hydroxamic acid derivative 6 (hereafter called RM-3-22) (Sharma et al., 2022). Notably, apart from acting on histones, HDACis are responsible for altering different target proteins (Zhao et al., 2014). In addition to being a potent HDACi in NSCLC, RM-3-22 has been previously shown to alter the cell death pathways, such as autophagy and apoptosis, and affect cell cycle progression, which we reported earlier (Sharma et al., 2022). However, the detailed molecular mechanism and signaling cascades through which RM-3-22 promotes cell death have not been explored yet.

In this study, we report for the first time that along with the prohibition of lung cancer, RM-3-22 also exhibits its anticancer effect on oral carcinoma, leukemia, and breast cancer. However, it shows significant potential for lung carcinoma. Again, it is shown to act better than SAHA and TSA, particularly in A549 cells in some cases. Notably, it does not exhibit toxicity toward the normal breast epithelial cell line MCF 10A and the mouse fibroblast cell line L929 (Sharma et al., 2022), further proving its usefulness as a promising candidate and a good choice for overcoming existing limitations in HDAC inhibitor-based therapies. Subsequently, the current study was aimed at the anticancer effects of the molecule on A549 cells *in vitro* and *in vivo*. Above all, we report the cascade of signaling created by RM-3-22 exposure.

Importantly, we observed that RM-3-22 can induce the expression of ferritin heavy chain 1 (FTH1), a protein that helps maintain the iron balance (Luo et al., 2024) by converting iron into a non-reactive form, thus mitigating oxidative stress. FTH1 acts as a tumor suppressor in NSCLC through the modulation of iron metabolism and the apoptotic pathway. It has been reported that FTH1 can regulate angiogenesis in endothelial cells during oxidative stress (Tesfay et al., 2012) and activate the function of P53 in NSCLC (Ali et al., 2021). The study of FTH1 is becoming a promising target of NSCLC because of its function as a tumor-suppressor gene (Ali et al., 2021; Biamonte et al., 2018). Therefore, our molecule can also suppress NSCLC by inducing this tumor-suppressor gene, representing a novel finding.

In summary, this study reports that RM-3-22 modulates PI3K/Akt/mTOR signaling, which is also a promising zone for drug development research. Its modulation further reflects other pathways such as autophagy, which further causes apoptosis and results in cell death.

## 2 Material and methods

### 2.1 Cell culture, growth conditions, and treatments

A549 was obtained from NCCS. A549 was grown in RPMI media (Sigma) supplemented with 10% fetal bovine serum (Gibco, US). Cells were cultured at 37°C temperature, 95% humidity, and 5% CO_2_ gas environment. In addition, MCF 10A, SiHa, FaDu, MCF7, T-47D, ZR-75-30, and K-562 cell lines were used in this study. MCF 10A, FaDu, and K-562 were purchased from ATCC and cultured in the recommended media and supplements by ATCC. For the MCF 10A culture, we used MEBM media (ATCC) supplemented with the MEGM kit (ATCC) and 100 ng/mL of cholera toxin (Sigma). SiHa was purchased from NCCS and was grown in EMEM media (Gibco). MCF7, T-47D, and ZR-75-30 were purchased from Sigma and grown in specific conditions and media recommended by Sigma. All the cell lines used in this work were between passage numbers 2 and 9. Every experiment was conducted from 65% to 70% confluent cells. Routine *mycoplasma* testing of each cell line was carried out in the laboratory using the conventional DAPI (4′,6-diamidino-2-phenylindole) nuclear staining method.

For the experimental purpose, 3-methyl adenine (3-MA) and rapamycin were added 1 h prior, and insulin was administered 30 min before RM-3-22 treatment. In every experiment, SAHA (Selleck) was used as the standard. TSA (Adooq Bioscience) was also used in some experiments.

### 2.2 Synthesis of RM-3-22

Our research group described the synthetic route of RM-3-22 synthesis earlier (Sharma et al., 2022). In brief, tetrahydroazepino quinazolinone-based hydroxamic acid was synthesized via a multi-step synthetic route. The reaction sequence involved the condensation of nitro-substituted anthranilic acid with caprolactam to produce fused quinazoline. The target compound was synthesized through a sequence of reactions including nitro reduction, benzylation with (E)-methyl 3-(4-(bromomethyl) phenyl) acrylate, ester hydrolysis, amidation with NH_2_OTHP, and TFA-mediated deprotection. The synthetic route is presented in the Supplementary Material. For the *in vitro* study, RM-3-22 was dissolved in DMSO. The final concentration of DMSO was <0.1% in all *in vitro* experiments. For the *in vivo* study, RM-3-22 was administered in mice with carboxymethylcellulose.

### 2.3 Reagents and chemicals

All the primary and secondary antibodies for Western blotting were obtained from Santa Cruz Biotechnology and Cell Signaling Technology (Supplementary Table S1). For immunofluorescence microscopy, Alexa Fluor 488 anti-rabbit was purchased from BioLegend. Every antibody was used according to the manufacturer’s recommended dilution. All the biochemicals and reagents were of molecular biology grade and purchased from Sigma-Aldrich. 3-MA (T1879) was purchased from TargetMol, and rapamycin (S1039) was purchased from Selleckchem. Ferritin heavy chain siRNA (h) (sc-40575) was purchased from Santa Cruz Biotechnology.

### 2.4 Cell proliferation assay

The MTT (3-(4,5-dimethylthiazol-2-yl)-2,5-diphenyl-2H-tetrazolium bromide) assay was carried out to check the cell viability. In brief, A549 cells were seeded in 96-well plates and treated with RM-3-22, SAHA, and TSA at various concentrations (5 nM–50 μM) for 24 h, 48 h, and 72 h, with or without 3-MA and rapamycin, depending on the experiment design. MTT was added 3 h before the termination time. Optical densities (ODs) were then measured at 570 nm using a multimode plate reader (Envision) (Guru et al., 2015).

### 2.5 Acridine orange staining

A549 cells were incubated in 6-well plates and treated with RM-3-22 at different doses and SAHA at a 0.5 μM dose for 24 h. Cells were then incubated with acridine orange (1 μg/mL) for 20 min and washed once with ice-cold PBS. Autophagy was determined by red fluorescence under a fluorescence microscope (ZEISS Axiocam 202 mono) (Kumar et al., 2015).

### 2.6 Flow cytometry analysis of apoptosis and necrosis

A549 cells were treated with RM-3-22 at concentrations of 0, 0.5, 1, 5, and 10 μM and SAHA at 0.5 μM concentrations for 24 h. Cells were stained using an Annexin/PI kit, following the manufacturer’s protocol. FACS analysis was carried out as described earlier (Kumar et al., 2013).

### 2.7 Measurement of mitochondria membrane potential

A549 cells were exposed to RM-3-22 at concentrations of 0, 0.5, 1, 5, and 10 μM and SAHA at 0.5 μM for 24 h. Mitochondria membrane potential (MMP) was then analyzed by flow cytometry using rhodamine 123 dye, following a previously established protocol (Guru et al., 2015).

### 2.8 DNA agarose gel electrophoresis

A549 cells were treated with RM-3-22 at different concentrations and SAHA for 24 h. After that, apoptosis was assayed by gel electrophoresis of the extracted genomic DNA contents, following a previously established protocol (Shashi et al., 2006).

### 2.9 Cell cycle analysis

Cell cycle analysis was carried out by propidium iodide fluorescence, as described previously (Kumar et al., 2015), using BD FACSVerse™ and analyzed using BD FACSuite flow cytometry software.

### 2.10 DAPI staining

In accordance with the experimental design, A549 cells treated or untreated with RM-3-22 were seeded in a 12-well plate and treated with RM-22. Following trypsinization, cells were washed in PBS, fixed with methanol–acetic acid, and evenly distributed on slides. After 8 min of DAPI staining (1 μg/mL), the samples were cleaned and mounted using coverslips. Finally, nucleus staining was identified using a fluorescence microscope (Zeiss Axiocam 202 mono) (Pathania et al., 2013).

### 2.11 Scratch assay

A549 cells treated or untreated with RM-3-22 were seeded in a 6-well plate according to the experimental condition. After 24 h, uniform scratches were made and photographed under a microscope. After 24 h, photographs were taken again, and the width of the scratches was measured using ImageJ software. The percentage of wound healing was calculated as follows: % wound closer = (0 h scratch width−24 h scratch width)/0 h scratch width*100. The migration capacity of the untreated control group was considered to be 100%. Considering that fact, the ability of the other groups was calculated (Guru et al., 2015).

### 2.12 Immunofluorescence confocal microscopy for LC3B detection

A549 cells were cultured on the sterile coverslips placed on the surface of 6-well plates at a density of 10,000 cells per well. Cells were treated with different concentrations of RM-3-22 (0.5–10 μM), with or without rapamycin, and 3-MA for 24 h. Then, the cells were fixed with 4% paraformaldehyde for 10 min at room temperature and permeabilized using 0.5% Triton-X in PBS for 5 min. The cells were blocked with 3% BSA for 20 min at room temperature. After that, cells were incubated overnight at 4°C with the MAP1LC3B primary antibody, diluted 1: 200 in 3% BSA in PBT. The next day, cells were again incubated with Alexa Fluor 488 conjugated anti-rabbit secondary antibody (406416 BioLegend) for 1 h (dilution 1:500) at room temperature in the dark, followed by washing with PBS. The coverslips were further counterstained with DAPI to mark the nuclei, and photographs were taken using the Leica DMi8 confocal imaging system (Guru et al., 2015).

### 2.13 Spheroid formation

Spheroids of A549 cells were generated in 96-well ultra-low attachment plates using B27 supplements (Thermo Fisher Scientific), EGF (PeproTech), and bFGF (PeproTech), following a previously described protocol (Li et al., 2019). After that, spheroids are treated with RM-3-22 (10 μM) for 24 h, 48 h, and 72 h with or without rapamycin and 3-MA depending on the experimental design.

### 2.14 RT-PCR analysis

A549 cells were treated with RM-3-22 (0.5–10 μM) and SAHA (0.5 μM) with or without rapamycin and 3-MA for 24 h. Additionally, A549 cells were cultured in monolayer (2D) and spheroid (3D) forms and were exposed to RM-3-22 (10 μM) for 24 h, 48 h, and 72 h. Subsequently, total RNA was extracted using TRI-Reagent (T9424 Sigma). After that, following the manufacturer’s instruction, cDNAs were synthesized from an equal amount of RNA (1 μg) using the Revert-Aid cDNA Synthesis Kit. SYBR Green PCR amplification was performed using the QuantStudio™ 7 Pro Real-Time PCR System. Two sets of primers, as listed in Supplementary Table S2, were used to amplify necessary genes (Guru et al., 2015).

### 2.15 Tumor xenograft experiment

All animals were matured at the Central Animal House facility, NIPER Hyderabad, with prior approval of IAEC (approval no. NIP/20200/PC/476). The NOD-SCID mice were purchased from Hylasco Laboratory and maintained as per the IAEC protocol. They were housed in an IVC system with a controlled environment with a temperature of 20–25°C, humidity of 50%–60%, and 12-h light/dark cycle. All the animals were provided free access to a standard chow diet (gamma-irradiated) with autoclaved drinking water. Before the onset of the study, the animals were endorsed to be acquainted with the experimental circumstances for a week. A total of 1.5 × 10^6^ A549 cells were mixed in PBS and diluted with Matrigel at a 1:1 ratio, followed by inoculation at the left flank of each animal. The mice were periodically checked for the tumor volume and the animals’ health. After 3 weeks, when the average tumor volume in the mice reached up to 100 mm^3^, all the mice were randomly divided into two groups. One group received treatment with RM-3-22 at a dose of 20 mg/kg orally using gavage five times with a three-day gap for three animals, while the three others received no treatment. After 2 weeks, all the animals were euthanized humanely in a carbon dioxide chamber (Zhao et al., 2018).

### 2.16 Histopathological evaluations of tumor xenograft

After the mice were euthanized, the tumor tissues were collected and immediately fixed in formalin (10% v/v). After 7 days, the tissues were dehydrated with increasing concentrations of ethanol and then embedded in paraffin. Sections of 5-μm thickness were prepared from the paraffin-embedded tissues using a microtome (LEICA RM2255).

To observe cellular morphology, the sections were deparaffinized with xylene, rehydrated again with decreasing concentrations of ethanol, and stained with hematoxylin and eosin (H&E) (Singla and Jena, 2022; Pai et al., 2018). After staining, the sections were covered with DPX mounting media and observed under the microscope (LEICA ICC50 W).

To observe particular protein localization, the VECTASTAIN^®^ Elite^®^ ABC Universal PLUS Kit (VectorLabs, catalog no. PK-8200) was used. After deparaffinization and rehydration, the cross-links between the fixative and epitopes of the proteins in the sections were disrupted by heating in citrate buffer (pH = 6). This was followed by endogenous peroxidase blocking and treatment with normal horse serum. Next, the sections were incubated overnight with the primary antibody, and after washing, they were again incubated with the biotinylated secondary antibody for half an hour. For the development of an antibody signal, a 3,3′-diaminobenzidine (DAB) solution was used, and the nucleus was counterstained with hematoxylin. All the images were quantified using ImageJ software according to the protocol described by Zhao et al. (2018).

### 2.17 Preparation of cell lysate and Western blot assay

A549 cells were subjected to RM-3-22 at earlier mentioned doses (0.5–10 μM) for 24 h and SAHA at 0.5 μM as a positive control with or without rapamycin, 3-MA, and insulin. Additionally, 5 μM of RM-3-22 was treated for 3 h, 6 h, 10 h, 18 h, and 24 h. Whole-cell, mitochondrial, and cytosolic fractions were prepared using the method described earlier (Guru et al., 2015). For *in vivo* sample preparation, tissues were first chopped into small pieces, then homogenized using a hand homogenizer, and further processed like the cellular samples. An equal amount of protein (40–70 μg) was loaded for SDS-PAGE and transferred to the polyvinylidene difluoride (PVDF) membrane. The membrane was blocked with 5% BSA in TBST and probed with primary and secondary antibodies, as mentioned in Supplementary Table S1.

### 2.18 Transfection of small interfering RNA

Human-specific FTH1 siRNA was transfected into A549 cells in accordance with the manufacturer’s instructions (Santa-Cruz, sc-40575). In short, cells were treated for 8 h with transfection media containing the transfection reagent and siRNA, and then they were added to full media for 48 h. Using Western blotting, the effectiveness of protein expression knockdown was evaluated (Kumar et al., 2013).

### 2.19 Bioinformatics analysis

#### 2.19.1 Dataset

Gene expression analysis of lung cancer patients was carried out based on The Cancer Genome Atlas (TCGA) breast cancer dataset. For survival analysis, TCGA gene expression data were analyzed using the Kaplan–Meier Plotter (Győrffy, 2021), and overall survival (OS) was analyzed using the median cut-off method. For checking the expression pattern change of specific genes from normal to lung adenocarcinoma patients, the UALCAN (https://ualcan.path.uab.edu/) (Chandrashekar et al., 2022; Chandrashekar et al., 2017) database was used. Gene correlation plots were adapted using CANCERTOOL (http://genomics.cicbiogune.es/CANCERTOOL) (Cortazar et al., 2018) using lung cancer datasets.

#### 2.19.2 Construction of protein–protein interactions

Genes of interest were searched in the search tool of the STRING database (https://string-db.org/) to identify the protein–protein interactions (PPIs). The network was then established using Cytoscape 3.9.0 software (The Cytoscape Consortium, United States).

#### 2.19.3 Molecular docking study

The structure of proteins is downloaded from the Protein Data Bank (PDB) (https://www.rcsb.org/search) and checked for broken chains and errors using the Discovery Studio Visualizer 3.0 (https://discover.3ds.com/discovery-studio-visualizer- download). Ligand structures were designed and drawn using the ChemDraw tool in ChemOffice-16 software (https://www.cambridgesoft.com/Ensemble_for_Chemistry/details/Default.aspx?fid=16). The selected chemical compounds and protein structures were uploaded to the virtual screening software interface PyRx (https://pyrx.sourceforge.io/) and AutoDock Vina (https://vina.scripps.edu/). The docking output files were analyzed for the interaction between the compound and the amino acid of the protein using the Discovery Studio Visualizer 3.0.

### 2.20 Statistical analysis

All the statistical work was carried out using GraphPad Prism 8.0. Data are expressed as the means ± SD. Student’s t-test was employed in some experiments. In addition, Dunnett’s method was employed for the one-way ANOVA test. A p-value of < 0.05 was considered statistically significant, with significance levels denoted as follows: *p < 0.05, **p < 0.01, and ***p < 0.001. The reported p-values associated with TCGA dataset analysis were obtained using the online portal.

## 3 Results

### 3.1 Cytotoxic profile of RM-3-22

**FIGURE 1:**
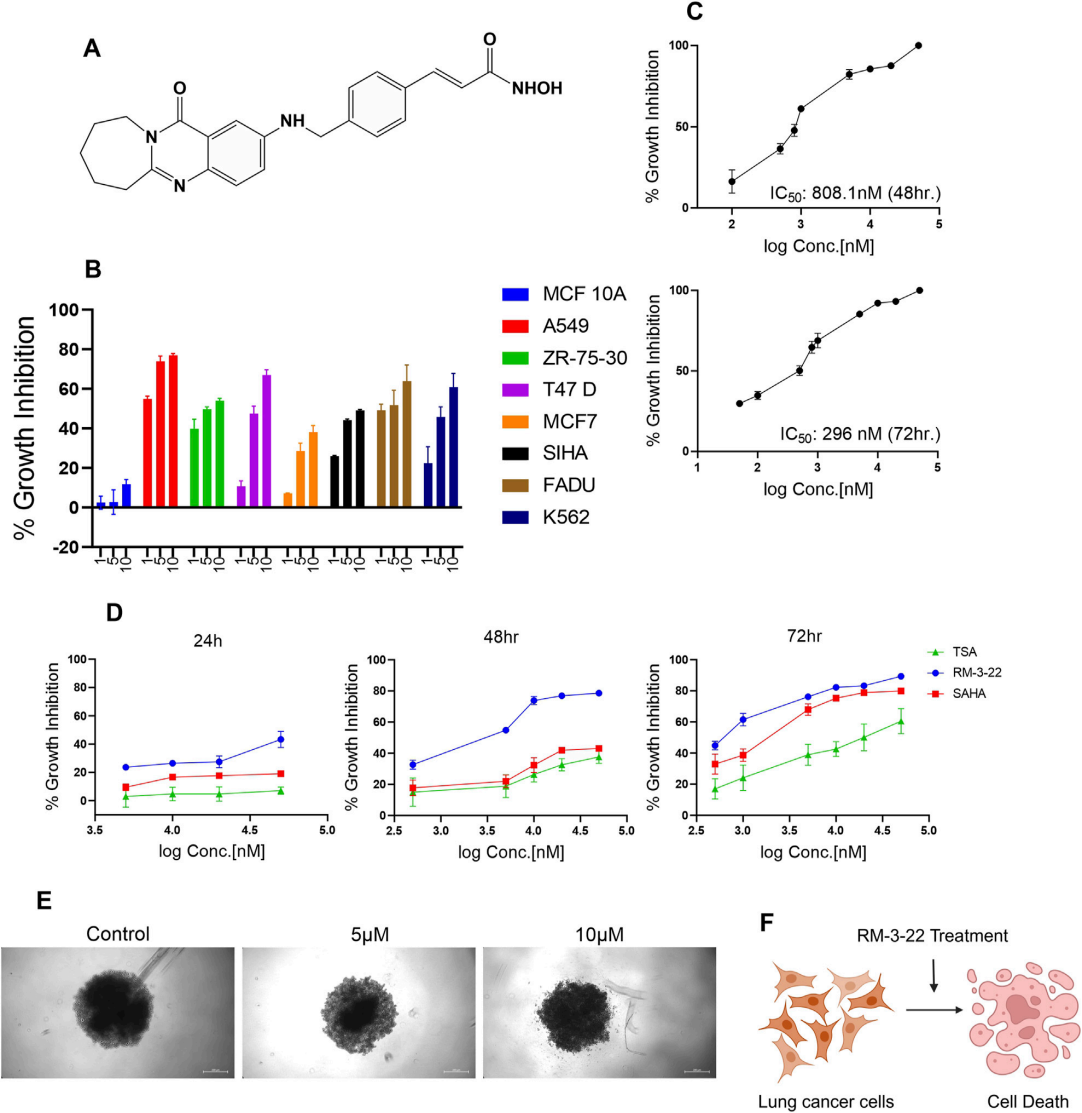
RM-3-22 induces the cytotoxicity of different cancer cell lines, especially the lung cancer cell line A549. **(A)** Chemical structure of RM-3-22. **(B)** RM-3-22 has cytotoxicity effect on different cancer cell lines, most effectively on A549, and does not affect MCF 10A. **(C)** Time-dependent IC_50_ curve of RM-3-22 against A549 cells. **(D)** Comparative time-dependent activity of TSA, SAHA, and RM-3-22 shows the superiority of RM-3-22. **(E)** Bright field image of A549 spheroid treated with different concentrations of RM-3-22 (scale bar = 200 µm). **(F)** Schematic representation summarizing the activity of RM-3-22 as an anti-lung-cancer agent.

To evaluate the effect of RM-3-22 (Figure 1A), we introduced this molecule to different cancer cell lines, such as the lung (A549), breast (ZR-75-30, T47 D, MCF7), cervical (SiHa), oral (FaDu), leukemia (K-562), and normal breast epithelial cell lines (MCF 10A) for 48 h. Among the other tumor cell lines, the A549 population showed the highest cell growth inhibition, leaving minimal effect on the normal cell line (Figure 1B) (Table 1). For further evaluation of the impact of RM-3-22, IC_50_ values of this agent were calculated for 48 h and 72 h. The IC_50_ values were 808 nM and 296 nM for 48 h and 72 h, respectively (Figure 1C). Interestingly, a time- and dose-dependent comparative analysis between RM-3-22 and known HDAC inhibitors “SAHA” and TSA depicted that RM-3-22 outperformed SAHA in potency (Figure 1D). This compound was also shown to distort the spheroidal structure of the A549 population after 72-h treatment at 5 μM and 10 μM doses (Figure 1E). These results suggest that RM-3-22 is a non-toxic therapeutic agent that could be used for therapeutic purposes. The summary of these results is portrayed in Figure 1F.

**TABLE 1:** IC_50_ values of RM-3-22 in different cell lines (^a^ SD, standard deviation; all experiments were performed independently at least three times).

Cancer type	Cell lines	IC_50_, 48 h μMa
Breast mammary normal	MCF 10 A	>100 μM
Lung cancer	A549	0.808 ± 0.18
Breast cancer	ZR-75-30	10.2 ± 2.01
Breast cancer	T47 D	5.1 ± 0.45
Breast cancer	MCF7	>100 μM
Cervical cancer	siHa	60.5 ± 2.51
Oral cancer	FaDu	2.6 ± 0.81
Myeloid leukemia	K-562	5.8 ± 0.91

### 3.2 RM-3-22 restricts the PI3K/Akt/mTOR pathway

**FIGURE 2:**
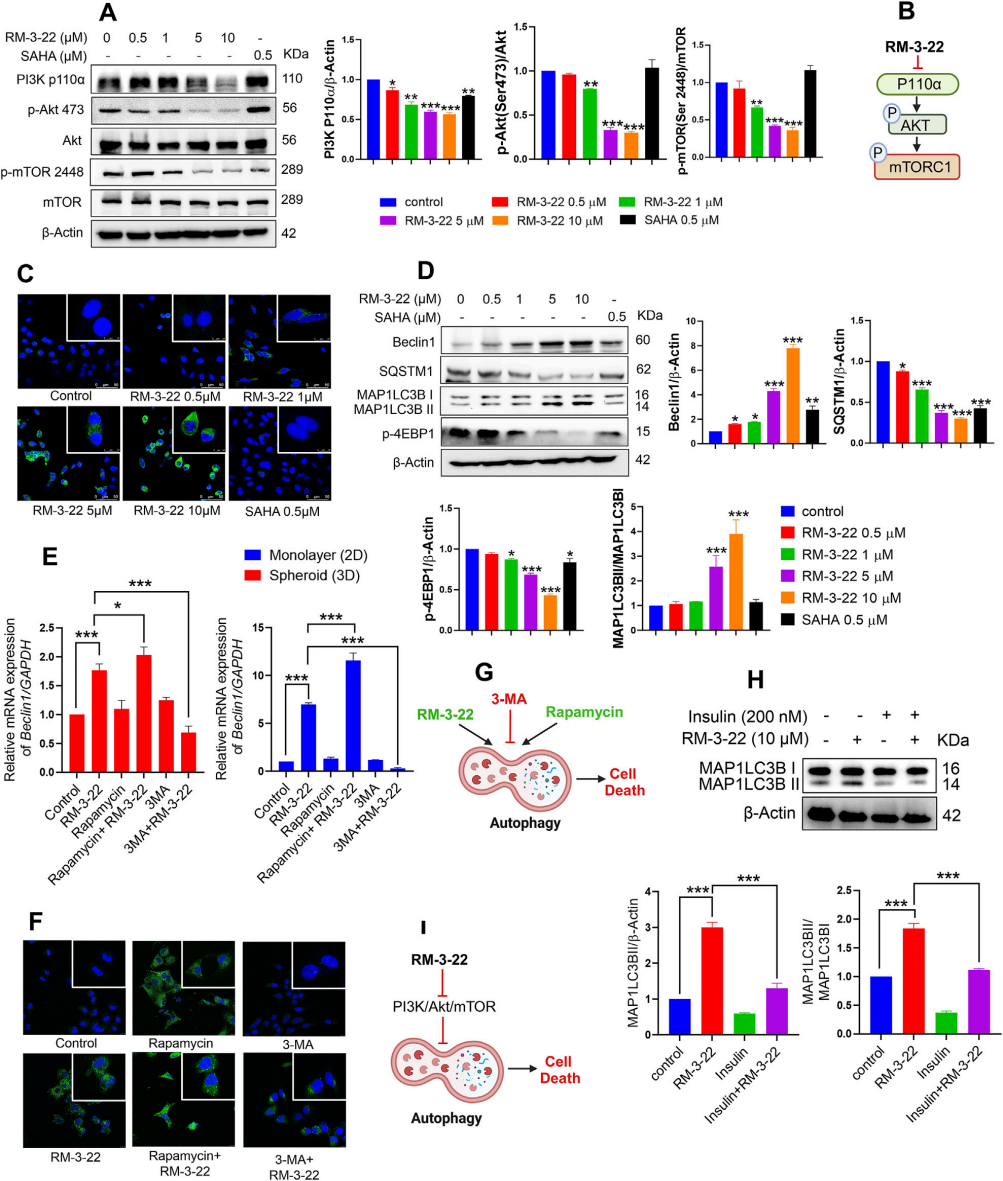
RM-3-22 induces autophagy pathway through the inhibition of PI3K/Akt/mTOR. **(A)** RM-3-22 inhibits the PI3K/mTOR/Akt pathway in a dose-dependent manner. Analyzed data shown are the means ± SD from three independent experiments and were analyzed using one-way ANOVA (*p < 0.05, **p < 0.01, and ***p < 0.001). **(B)** Schematic representation summarizing the PI3K/mTOR/Akt inhibition by RM-3-22. **(C)** Immunofluorescence of MAP1LC3B was assessed in A549 cells treated or untreated with RM-3-22 and SAHA. Images were taken at ×63 magnification. They show the enhancement of LC3B puncta due to exposure to RM-3-22 in a dose-dependent way. **(D)** Essential autophagy-related proteins such as Beclin1, SQSTM1, MAP1LC3B, and p-4EBP1 were checked by Western blot. Data represent the mean ± SD of three independent experiments (one-way ANOVA; *p < 0.05, **p < 0.01, and ***p < 0.001). **(E)** A549 cells in monolayer (2D) and spheroid (3D) forms were treated with or without RM-3-22 (10 μM), rapamycin (100 nM), and 3-MA (5 mM). Beclin1 increased in the mRNA level both in 2D and 3D configurations of A549 cells. Enhancement of autophagy subsequently increases the effect of RM-3-22, and the inhibition of autophagy again decreases RM-3-22’s effect. Data represent the mean ± SD on three independent experiments (one-way ANOVA; *p < 0.05, **p < 0.01, and ***p < 0.001) for control, rapamycin, rapamycin + RM-3-22, 3-MA, and 3-MA + RM-3-22 vs. RM-3-22. **(F)** MAP1LC3B puncta formation is exhibited to be enhanced due to the additive action of rapamycin with RM-3-22 and decreased due to the antagonistic effect of 3-MA compared to the sole treatment with RM-3-22 in A549 cells. Images were taken at ×63 magnification. **(G)** Schematic representation summarizing the figure of autophagy induction by RM-3-22. **(H)** Application of well-known PI3K/Akt/mTOR pathway-inhibitor insulin in the presence or absence of RM-3-22 leads to the inhibition of autophagy induction. The data shown are the mean ± SD from two independent experiments and were analyzed using one-way ANOVA (*p < 0.05, **p < 0.01, and ***p < 0.001) for control and insulin + RM-3-22 vs. RM-3-22. Schematic diagram of the summary is represented in **(I)**.

The well-known comprehension is that the PI3K/Akt/mTOR pathway plays a crucial role in cancer growth and tumor proliferation. The inhibition of the PI3K/Akt/mTOR pathway leads to the induction of autophagy and apoptosis. This study reports a novel finding that RM-3-22 causes PI3K/Akt/mTOR pathway inhibition in A549 cells. Notably, RM-3-22 impeded all the significant proteins connected to the PI3K/Akt/mTOR pathway, such as PI3K P110α, p-Akt (Ser473), and p-mTOR (Ser2448). This outcome was confirmed through Western blot analysis (Figure 2A). The summary of this finding is represented in Figure 2B.

### 3.3 RM-3-22 encourages the upregulation of the autophagic pathway

Nowadays, autophagy, a type-2 programmed cell death, has evolved as a highly investigated field within cancer biology. In addition, SAHA also exhibited the induction of autophagy in lung cancer cell lines. We commenced an exploration of potential functional similarities in the context of autophagy between these two compounds as we previously reported that RM-3-22 also works as an HDAC inhibitor. Strikingly, RM-3-22-treated A549 cells exhibited robust initiation of autophagy.

RM-3-22-induced autophagy was demonstrated by acridine orange staining of A549 cells (Supplementary Figure S2A). To provide additional vindications, we performed qRT-PCR analyses of A549 cells cultured in 2D configurations under different doses of RM-3-22 administration, monopolizing with essential genes within autophagy (Supplementary Figure S2B). This observation was further reinforced using a MAP1LC3B-specific immunofluorescence assay, which revealed that macroscopic M+AP1LC3B puncta increased in a dose-dependent manner of the molecule (Figure 2C). Autophagy induction was further confirmed by Western blot analysis of crucial autophagy proteins such as SQSTM1 (P62), Beclin1, MAP1LC3B, and P-4EBP1 (Figure 2D). The downregulation of SQSTM1 and the upregulation of the other mentioned proteins confirmed the induction of autophagy flux by the molecule as autophagosome degradation by lysosome decreases the level of SQSTM1 during autophagy.

Moreover, RM-3-22 was administrated at 10 μM concentration in the 3D configuration of A549 cells for different time points. After that, qRT-PCR analysis of ATG7 and Beclin1 revealed the upregulation of autophagy in the 3D form of A549 cells due to RM-3-22 (Supplementary Figure S2CI,II). We used the autophagy inhibitor 3-MA and inducer rapamycin to check the additive effect (Supplementary Figure S2E). The administration of 3-MA (5 mM) in combination with RM-3-22 (10 μM) reduced the autophagy, whereas the combined effect of rapamycin (100 nM) and RM-3-22 increased the autophagy drastically. This was confirmed by checking Beclin1 expression in the mRNA level in 2D or 3D forms (Figure 2E). Furthermore, this proclamation was strengthened by the immunofluorescence data of the MAP1LC3BII protein, which further affirmed the autophagy induction property of RM-3-22 (Figure 2F).

Interestingly, rapamycin triggered cell morality and 3-MA rescued the mortality effect carried by RM-3-22, which was confirmed by the MTT assay (Supplementary Figure S2F). This result confirmed RM-3-22 and activation of autophagy by rapamycin, which induced the death of A549 cells. Moreover, Supplementary Figure S2E also depicts the enhancement of cell mortality by combining rapamycin and RM-3-22. Furthermore, the size of the spheroid was also reduced in the combination treatment (Supplementary Figure S2E), confirming the additive effect of rapamycin on RM-3-22 in A549 cells. Notably, the working doses of rapamycin and 3-MA exhibited no fatal effect on the viability of A549 cell populations, as verified by MTT assays conducted for 48 h duration of those with varying concentrations (Supplementary Figure S2D). The conclusive results are summed up in Figure 2G.

### 3.4 Induction of autophagy is caused by PI3K/Akt inhibition by RM-3-22

To investigate the possible regulation of autophagy by the PI3K/Akt pathway, A549 cells were pre-treated with 200 nM insulin, a well-known activator of this signaling pathway, 30 min before RM-3-22 treatment, with a total period of 24 h. Interestingly, samples that underwent insulin pre-treatment illustrated a reduction in the expression of MAP1LC3B-II compared to samples solely exposed to RM-22 (Figure 2H). This finding establishes a strong interdependency between PI3K/Akt activity and the distortion of autophagy in these circumstances (Figure 2I).

### 3.5 RM-3-22 significantly induces caspase-dependent apoptosis in A549 cells

**FIGURE 3:**
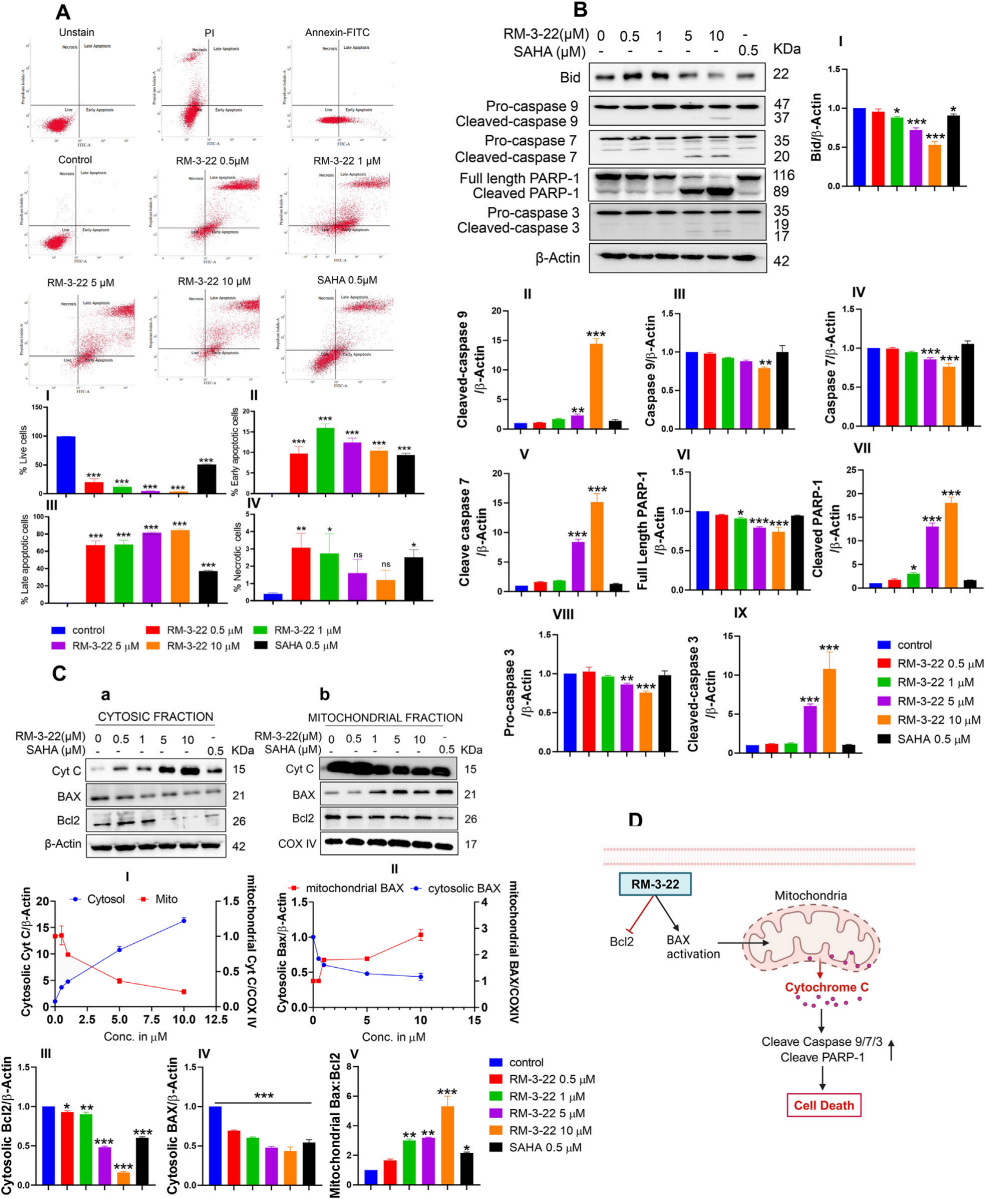
RM-3-22 induces apoptotic death in A549 cells. **(A)** Annexin V/PI staining shows the enhancement of the apoptotic population of cells along with an increase in the RM-3-22 concentration via flow cytometry. Analyzed data are represented in I, II, III, and IV. Data are presented as the mean ± SD of three independent experiments; *p < 0.05, **p < 0.01, and ***p < 0.001, compared with the untreated control, were considered statistically significant. **(B)** Western blot analysis of whole-cell lysate of apoptosis-related proteins such as BID, caspase 9, caspase 7, PARP-1, and caspase 3 was shown to confirm RM-3-22’s ability to induce apoptosis. Data represent the mean ± SD of three independent experiments (one-way ANOVA; *p < 0.05, **p < 0.01, and ***p < 0.001). **(C)** Cytosolic and mitochondrial fractionation was carried out to show the translocation of BAX and Cyt C from the cytosol to mitochondria and *vice versa*, respectively, and reduction in Bcl2 expression due to exposure to RM-3-22. Data represent the mean ± SD of three independent experiments (one-way ANOVA; *p < 0.05, **p < 0.01, and ***p < 0.001). Schematic representation summarizing the figure of induction of apoptosis by RM-3-22 is represented in **(D)**.

RM-3-22 exhibited its capacity to stimulate apoptosis in A549 cells, as confirmed by a series of experiments affirms that. The apoptotic potential of RM-3-22 was corroborated through Annexin/PI staining to differentiate between apoptotic and necrotic cell death. Significant apoptotic death was found in a low dose within 24 h, while necrotic death remained negligible (Figure 3A). Furthermore, exposure of RM-3-22 to A549 led to a remarkable decrease in mitochondrial membrane potential in a dose-dependent manner (Supplementary Figure S3A). Apoptosis was further verified by observing the fragmented DNA, caused by RM-3-22 exposure, in DNA agarose gel electrophoresis assay (Supplementary Figure S3B). To bolster this observation, the expression of anti-apoptotic protein Bcl2 was checked at mRNA level in A549 cells grown in 2D configurations and exposed to RM-3-22 in a dose-dependent manner. These assessments further substantiated the occurrence of apoptosis (Supplementary Figure S3C). Additionally, the activation of caspase 3 and PARP-1 cleavage in A549 cells due to RM-3-22’s dose-dependent exposure reaffirmed the occurrence of apoptosis. Along with that, Bid expression decreased in the whole-cell lysate. Moreover, caspase 7 and caspase 9 cleavage occurred due to RM-3-22 exposure (Figure 3B). Furthermore, the release of cytochrome C (Cyt C) from mitochondria to cytosol and the translocation of BAX from cytosol to the mitochondrial membrane, two significant events of apoptosis, were checked and confirmed by mitochondria and cytosolic fractionation after exposure to RM-3-22 at different doses for 24 h (Figure 3C). All these cumulative results indicated that RM-3-22 is a potent apoptosis inducer. The summary of these results is represented in pictorial form in Figure 3D.

### 3.6 RM-3-22 treatment causes cell cycle arrest in the G2/M phase by the disruption of cyclin B and CDC2

**FIGURE 4:**
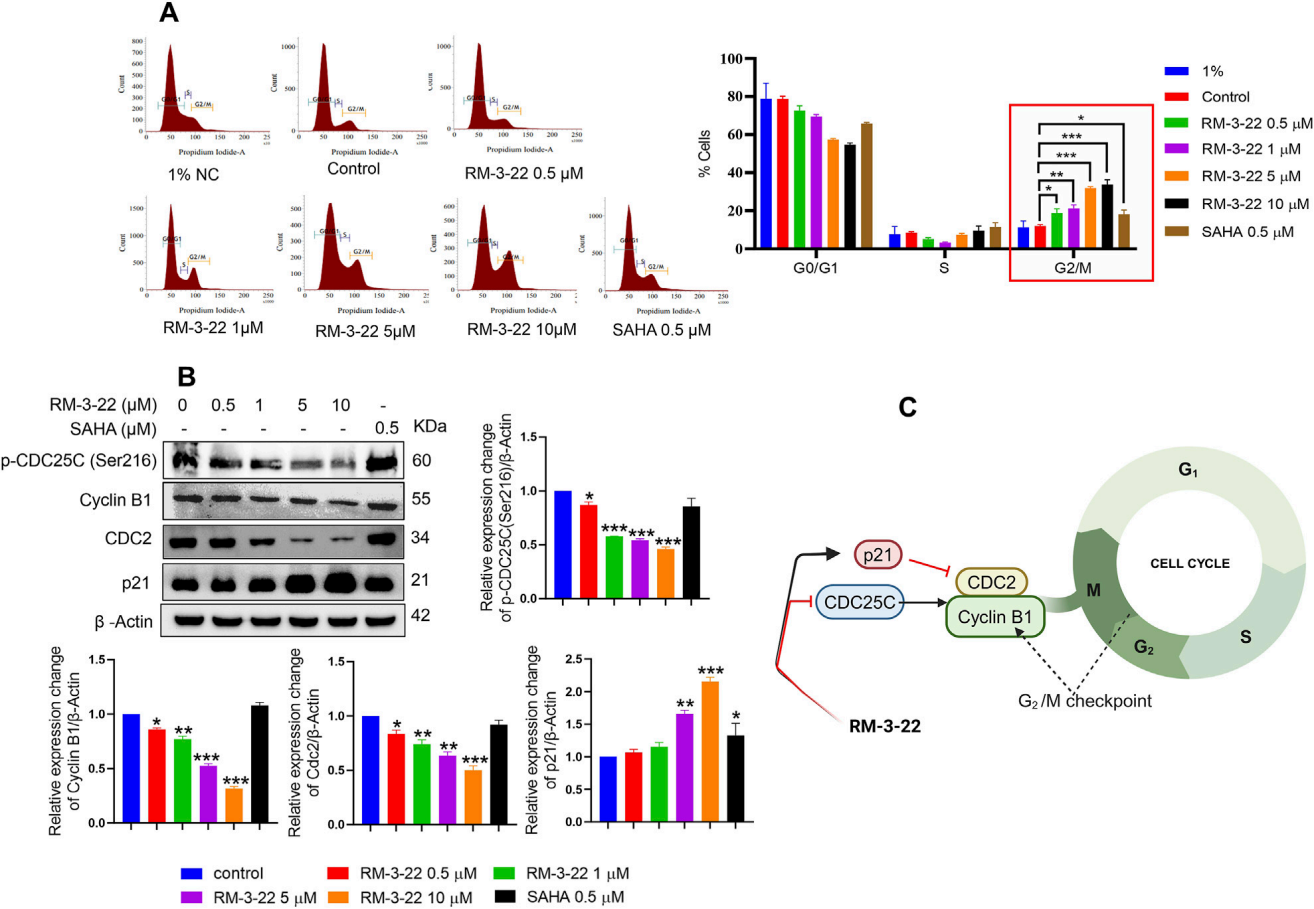
RM-3-22 induces G2/M arrest. **(A)** Flow cytometry of A549 cells treated or untreated with RM-3-22 and SAHA shows a greater number of cells in the G2/M phage in a higher concentration of RM-3-22. Data are presented as the mean ± SD of three similar experiments; p-value < 0.05 was considered to be significant, with *p < 0.05, **p < 0.01, and ***p < 0.001, compared with the untreated control in the G2/M phase. **(B)** Western blot for G2/M arrest-related proteins was checked after exposure to RM-3-22 and SAHA. Data represent the mean ± SD of three independent experiments (one-way ANOVA; *p < 0.05, **p < 0.01, and ***p < 0.001). **(C)** Schematic representation summarizing the figure of G2/M arrest by RM-3-22.

Earlier, we reported that RM-3-22 serves as the driver of G2/M phase arrest, as shown by flow cytometry analysis. In this section, we analyzed its arrestation activity, which increased in a dose-dependent manner (Figure 4A). A detailed molecular mechanism is outlined. From Western blot data (Figure 4B), a robust decrease in cyclin B1 and CDC2, the primary drivers of the G2/M transition, was noticed. This reduction was further reflected by the marked decrease in p-CDC25C (ser216) levels. Notably, a mentionable decrease in the expression levels of p21 was followed by RM-3-22 exposure. p21 acts as a critical cell cycle checkpoint regulator by inhibiting the activity of cyclin-dependent kinases (CDKs), a group encompassing those associated with the G2/M transition. This inhibition orchestrates the cell cycle halt. The comprehensive evaluation of these factors collectively suggests that RM-3-22 plays a role in inducing G2/M phase arrest. The summary of the results is represented in Figure 4C.

### 3.7 The initiation of autophagy caused by RM-3-22 plays a crucial role in the occurrence of apoptosis and G2/M arrest

Next, we aimed to thoroughly understand the interactions among distinct pathways, i.e., autophagy, apoptosis, and G2/M arrest, following exposure to RM-3-22.

**FIGURE 5:**
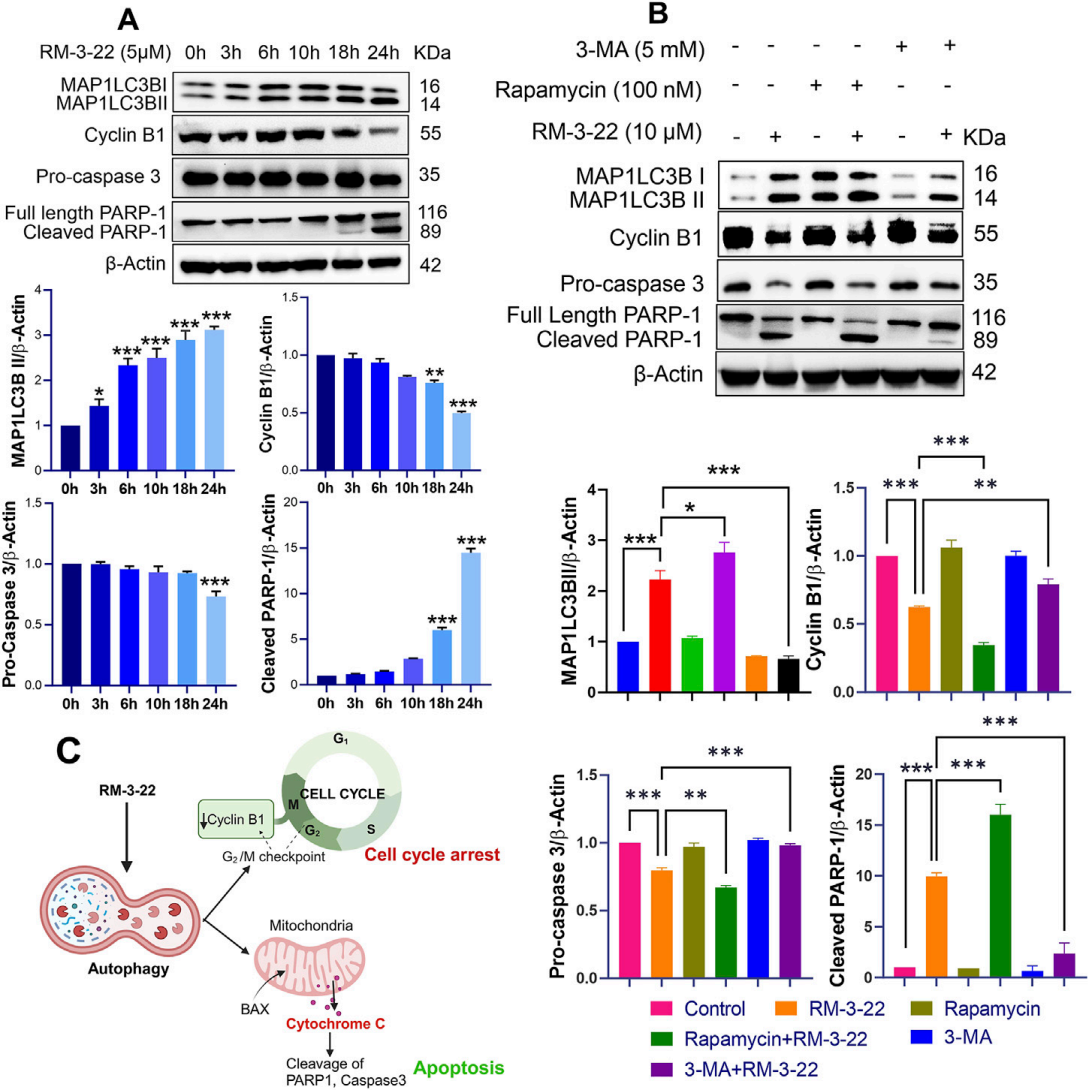
Onset of autophagy regulated the cell-cycle arrest and apoptosis upon RM-3-22 exposure in A549 cells. **(A)** Western blot analysis for MAP1LC3B, cyclin B1, pro-caspase-3, and PARP-1 was carried out to check time-dependent expression pattern change in the mentioned proteins upon exposure to RM-3-22. MAP1LC3B expression was initiated as early as 3 h after exposure to the compound. Cleavage of PARP-1 and caspase-3 began at 18 h, and expression of cyclin B1 decreased evidently at 18 h. Data represent the mean ± SD of three independent experiments (one-way ANOVA; *p < 0.05, **p < 0.01, and ***p < 0.001). **(B)** Western blot analysis using different inhibitors depicts that the pre-treatment of autophagy inducer rapamycin with RM-3-22 synergically induces the lipidation of MAP1LC3BII and PARP-1 cleavage and also downregulates the pro-caspase-3 and cyclin B1. Pre-treatment of 3-MA in A549 cells with RM-3-22 shows the exact opposite effect of rapamycin. Data represent the mean ± SD of three independent experiments (one-way ANOVA; *p < 0.05, **p < 0.01, and ***p < 0.001) for control, rapamycin, rapamycin + RM-3-22, 3-MA, and 3-MA + RM-3-22 vs. RM-3-22. Summary is represented in **(C)**.

Importantly, we conducted an extensive analysis to identify the concentration point at which the onset of the studied major protein expression, associated with the observed altered pathways upon exposure to various concentrations of RM-3-22, occurred. We found that all signaling’s events were significantly evident from the 5-μM concentration of RM-3-22, administered to A549 cells for 24 h. So, we checked the essential proteins responsible for autophagy (MAP1LC3B), apoptosis (PARP-1 and caspase 3), and G2/M arrest (cyclin B1) upon exposure to 5 μM RM-3-22 for varying durations of exposure. Our observations highlighted the primary initiation of MAP1LC3B lipidation after just 3 h of exposure to RM-3-22 in A549 cells (Figure 5A). In contrast, cyclin B1 degradation, reduction in pro-caspase 3 expression, and cleavage of PARP-1 were significantly identified after approximately 18 h of RM-3-22 exposure (Figure 5A). This evidence implies that autophagy was the earliest to be activated among the three pathways mentioned.

Furthermore, we tried to examine whether autophagy plays any role in the regulation of other pathways. To achieve this goal, we used the autophagy activator rapamycin and the inhibitor 3-MA in combination with RM-3-22. Combined treatment with rapamycin and RM-3-22 exhibited an increase in the expression of major autophagy-related proteins, MAP1LC3B-II, apoptosis-related proteins, cleave PARP-1, and a reduction in pro-caspase 3 and the G2/M arrest marker protein cyclin B1, compared to the values in A549 cells treated with RM-3-22 alone. Notably, the combined treatment of 3-MA and the molecule exhibited the reverse results of the previous one (Figure 5B). This result remarkably indicates that autophagy is the critical factor for the modulation of apoptosis and cell cycle arrest upon exposure to RM-3-22 (Figure 5B). The summary of these results is depicted in Figure 5C.

### 3.8 RM-3-22 enhances the expression of the tumor suppressor gene *FTH1*

**FIGURE 6:**
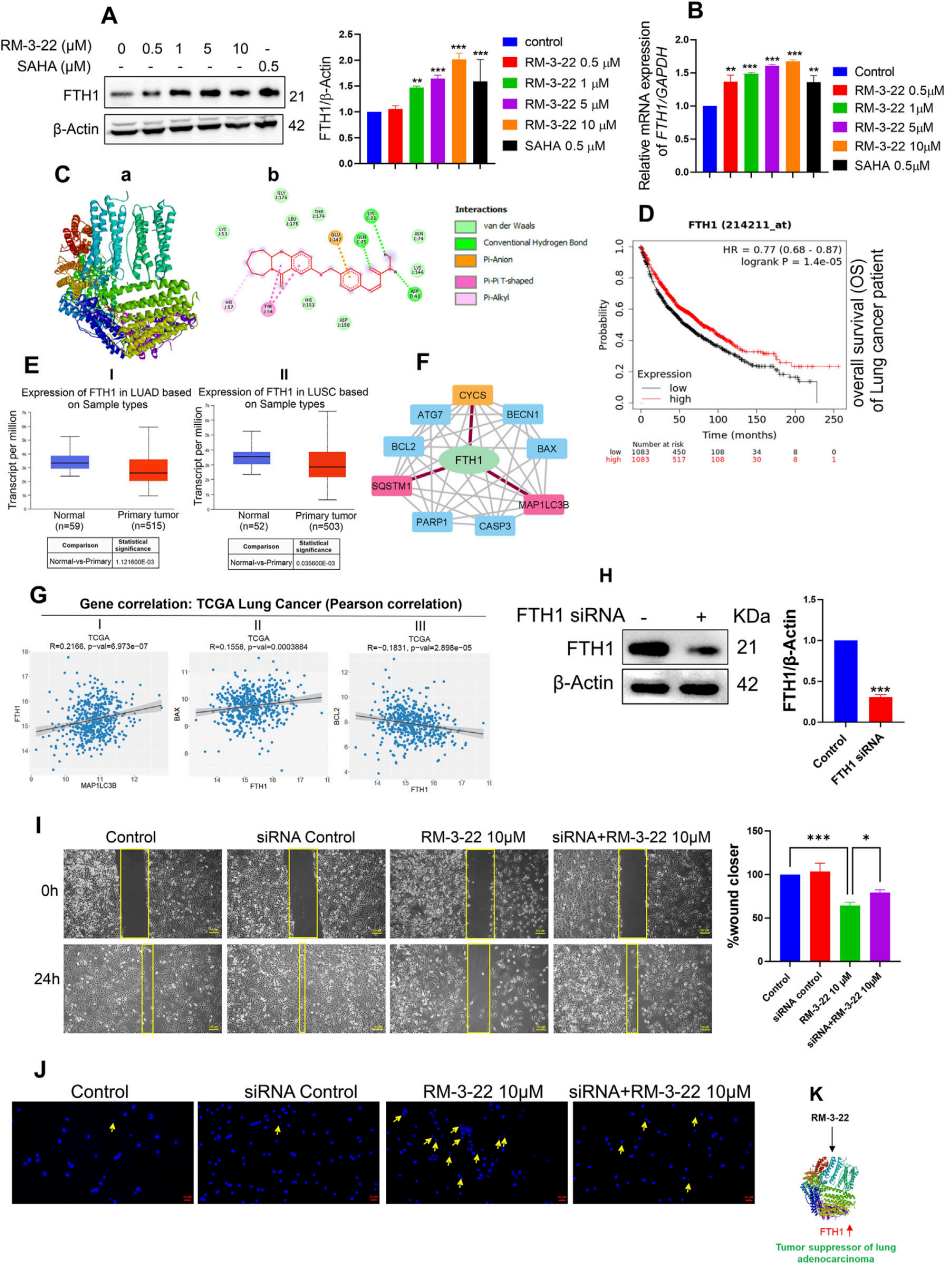
RM-3-22 induces the upregulation of FTH1, a tumor suppressor of NSCLC, which is positively correlated with apoptosis and autophagy pathway. **(A)** Western blot analysis shows the increased expression of FTH1. Data represent the mean ± SD of three independent experiments (one-way ANOVA; *p < 0.05, **p < 0.01, and ***p < 0.001). **(B)** qRT-PCR of FTH1 shows an increase in the mRNA level due to RM-3-22 exposure, both in a concentration-dependent manner. Data represent the mean ± SD of three independent experiments (one-way ANOVA; *p < 0.05, **p < 0.01, and ***p < 0.001). **(C)** Protein–ligand interaction diagram; (a) crystal structure of FTH-1 (PDB: 6J4A) interacting with ligand RM-3-22. (b) 2D molecular interaction of FTH-1 with RM22, docked using AutoDock Vina. Projected bonds are shown. Docking score -8.1. **(D)** Kaplan–Meier plotter survival analysis for patients with expression of FTH1. Survival analysis was performed in KM plotter using publicly available microarray data that are available within the database. A log-rank p-value ≤0.05 was considered significant. HR, hazard ratio. The red curve represents high expression, and the black curve represents low expression. OS)of the patients increases with high expression of FTH1 in lung cancer. **(E)** The FTH1 transcript level seems to decrease slightly during LUAD and lung squamous cell carcinoma (LUSC) progression in the patients (UALCAN database, TGCA sample), which indicates the tumor-suppressive nature of FTH1 in lung cancer. **(F)** PPI network of FTH1, autophagy, and apoptosis-related proteins constructed in Cytoscape based on interaction information derived from the STRING database. The network contains 10 nodes (proteins) and 37 edges (interactions). PPI showed the interaction of FTH1 with SQSTM1 and MAP1LC3B and FTH1 with CYCS (cytochrome C). For this specific interaction, edge color is specified as red. **(G)** Gene correlation analysis exhibited a positive correlation of FTH1 with MAP1LC3B and pro-apoptotic protein BAX and a negative correlation with anti-apoptotic protein Bcl2 at a significance level (*p < 0.05) (data from TCGA, cbiojune.en). **(H)** FTH1 knockdown by siRNA in A549 cells. Data represent the mean ± SD of three independent experiments (t-test; *p < 0.05, **p < 0.01, and ***p < 0.001). **(I)** Migration efficiency was higher in FTH1-downregulated cells treated with RM-3-22 compared to normal A549 cells solely treated with RM-3-22. **(J)** FTH1 knockdown led to the reversal of nuclear fragmentation, which is accompanied by RM-3-22. **(K)** Schematic representation showing the regulation of FTH1 by RM-3-22.

Our study unveiled that RM-3-22 induced the FTH’s expression in a dose-dependent manner in both protein levels (Figure 6A) and mRNA levels (Figure 6B), which was confirmed by Western blot and qRT-PCR analysis. The molecular docking study of RM-3-22 with *FTH1* exhibited PyRx binding scores as −8.1 (Figure 6C).

Notably, *FTH1* appears to play a controversial role in the progression of cancer. In myeloid leukemia (Zhang et al., 2023), hepatocellular carcinoma, Hodgkin’s lymphoma, head and neck carcinoma (Liao et al., 2023), and pancreatic cancer, *FTH1* has been shown to act as an oncogene. Meanwhile, in breast and lung carcinoma, it acts as a tumor suppressor gene (Lee et al., 2009; Ali et al., 2021). To further investigate the role of *FTH1* specifically in lung carcinoma, we used the TCGA km plotter and UALCAN database. From the TCGA KM plotter, we have discovered that the low expression of the *FTH1* level leads to poor survival in patients with lung carcinoma (Figure 6D). In addition, from the UALCAN database, we found a slightly decreased level of *FTH1* in the primary tumor of lung adenocarcinoma and lung squamous cell carcinoma compared to that in the normal one (Figure 6E). Based on these observations from the *in silico* study, we concluded the role of *FTH1* as a tumor suppressor gene in lung cancer.

### 3.9 FTH1 induction is correlated with apoptosis and autophagy

PPIs between FTH1, apoptosis, and autophagy-related proteins were analyzed using the STRING database and Cytoscape. The result exhibited a sort of interaction between FTH1 and Cyt C, as well as with SQSTM1 and MAP1LC3B (Figure 6F). This observation signifies the connection between FTH1 with autophagy and apoptosis. Correlation analysis using the cancer tool suggests that FTH1 has a positive correlation with MAP1LC3B and pro-apoptotic protein BAX, whereas it is negatively correlated with the anti-apoptotic protein Bcl2 in lung carcinoma (Figure 6G). This observation strengthens the previous observation.

### 3.10 FTH1 silencing leads to enhanced aggressiveness of A549 cells

To explore the role of FTH1 in this current context, we knocked down FTH1 through siRNA (Figure 6H). Furthermore, we treated the knockdown cells with 10 µM of RM-3-22 for 24 h and carried out a migration assay and DAPI staining. The scratch assay also exhibited that FTH1 knockdown cells treated with RM-3-22 had more migratory potential than RM-3-22-treated normal A549 cells (Figure 6I). Moreover, DAPI staining showed that RM-3-22-treated knockdown cells exhibited reduced nuclear fragmentation compared to RM-3-22-treated normal A549 cells. This proved that FTH1 knockdown led to the survival of A549 cells even after RM-3-22 treatment (Figure 6J). These data showed that FTH1 acts as a tumor suppressor in A549 cells and that RM-3-22-mediated enhancement of FTH1 expression could provide an additional therapeutic benefit (Figure 6K).

### 3.11 RM-3-22 suppresses tumor growth by inducing autophagy, apoptosis, cell cycle arrest, and FTH1 upregulation

**FIGURE 7:**
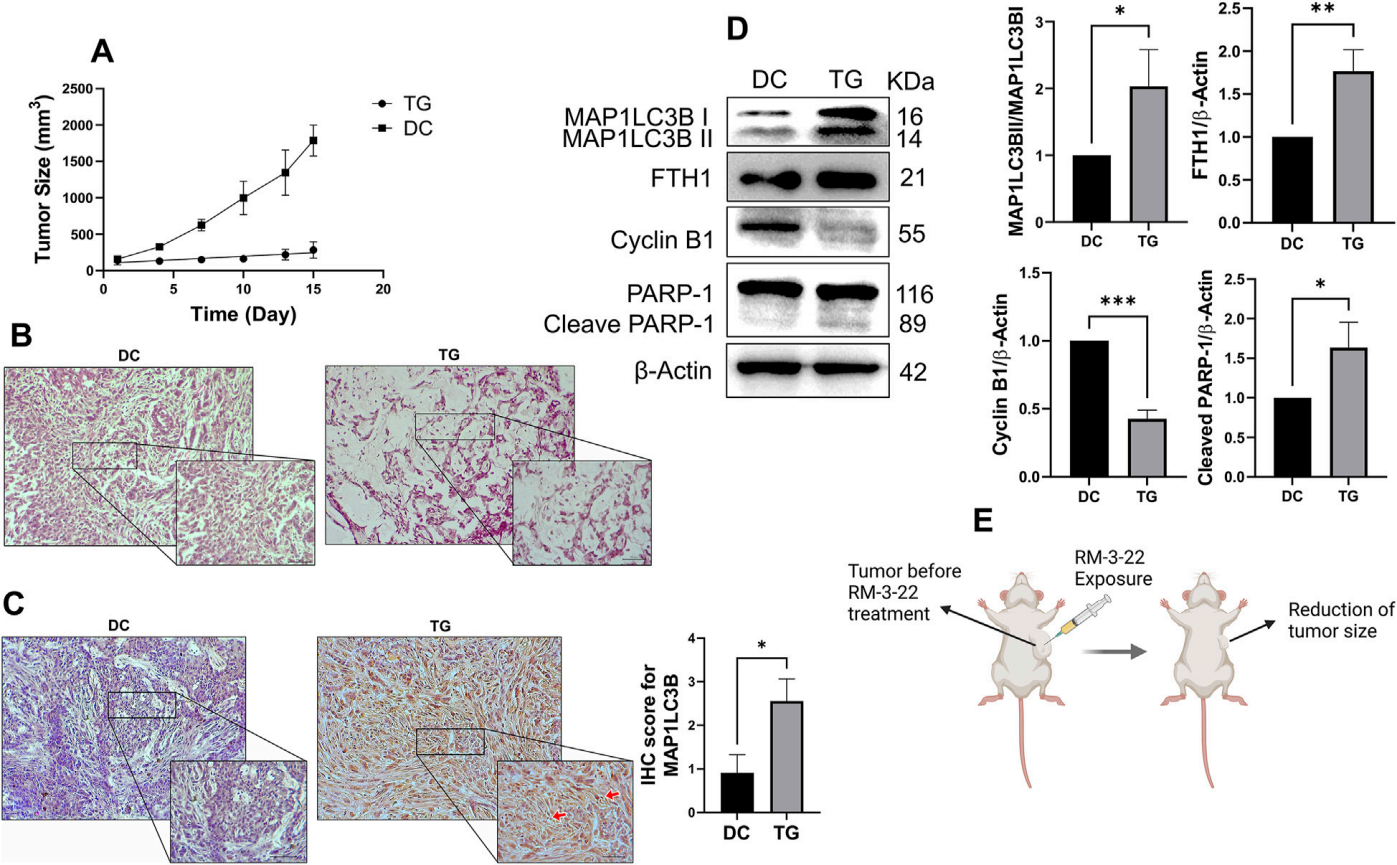
RM-3-22 suppresses tumor growth and induces autophagy, apoptosis, cell-cycle arrest, and FTH1 upregulation. **(A)** Tumor volume reduced in the treated group after treatment of RM-3-22 (DC, disease control; TG, treated group). Tumor sizes were measured every 2 days using the formula tumor volume= (width)^2^ x length/2. **(B)** Representative photomicrographs of hematoxylin and eosin staining showed disrupted tissue structure and damaged nuclei in RM-3-22-treated tissue. Scale bar = 100 μm (the box indicates the close-up view of disrupted structures; scale bar = 50 μm). **(C)** IHC of MAP1LC3B protein expression from DC and TG of mice. Scale bar = 100 μm (the box indicates the close-up view; scale bar = 50 μm). Data represent the mean ± SD of three independent experiments (t-test; *p < 0.05, **p < 0.01, and ***p < 0.001). **(D)** Western blot analysis was used to calculate the expression levels of MAP1LC3B, FTH1, cyclin B 1, and PARP-1 in tumor samples. Data represent the mean ± SD of three independent experiments (*p < 0.05, **p < 0.01, and ***p < 0.001). Schematic representation is presented in **(E)**.

The *in vitro* studies showed that RM-3-22 had an anti-cancer effect of suppressing lung cancer progression. *In vivo* experiments were performed in a mouse xenograft model treated with or without RM-3-22 for further confirmation. The administration of RM-3-22 markedly reduced the size of tumors (Figure 7A). Hematoxylin and eosin staining confirmed the traces of apoptotic cell-like structures in the treated tumor group (Figure 7B). IHC staining of MAP1LC3B indicated that its level was clearly increased in RM-3-22-treated mice (Figure 7C). In line with *in vitro* studies, RM-3-22 evidently induced the lipidation of MAP1LC3B and FTH1, prompted the cleavage of PARP-1, and reduced cyclin B1 expression (Figure 7D). Therefore, all these findings from *in vivo* studies suggested that RM-3-22 induced autophagy, apoptosis, cell cycle arrest, and FTH1 upregulation in the xenograft mice model. This compound can control tumor progression in mouse models (Figure 7E).

## 4 Discussion

Abnormality in any step of epigenetic regulation could alter the significance of gene expression, potentially leading to cancer. Among various epigenetic regulation patterns, histone deacetylation stands out as an essential mechanism closely related to the occurrence of cancer. Therefore, HDACis are often used for treating cancer (Xu et al., 2007). As per our previous study, one such HDACi, RM-3-22, potentially inhibits histone deacetylase activity in NSCLC (Sharma et al., 2022). However, the precise mechanism through which it restricts the cell cycle progression and triggers apoptosis of NSCLC remains unclear.

Unhindered cell division and proliferation are hallmark features of malignant tumors. So, evaluating the inhibitory index is the primary screening factor for selecting an anticancer drug. Through MTT assays, we have observed that RM-3-22 drastically reduces the cell populations of different cancers without harming the normal cell line. Notably, our previous study also showed that the compound has minimal toxicity in mouse fibroblast cells (L929) (Sharma et al., 2022). Importantly, among all screened cell lines, RM-3-22 performed most professionally in A549 cells. Our results showed that RM-3-22 is more effective than the HDAC inhibitors SAHA and TSA, which are currently available.

In this study, we have uncovered that our compound interferes with cell survival by activating two major cell-death pathways, namely, autophagy and apoptosis. The primary role of autophagy is to eliminate the damaged proteins and organelles and help in cell survival during stress. Under different conditions, however, autophagy may induce cell death. In cancer, it plays a dual role, both in tumorigenesis and as a tumor suppressor (Debnath et al., 2023). Autophagy is mediated by the inhibition of PI3K/Akt/mTOR (Peng et al., 2022). This pathway acts as a survival pathway found to be abnormally activated in different types of malignancies, which drives the progression of tumors (Rascio et al., 2021). Our data showed that RM-3-22 suppressed the expression of P110α, the active form of Akt, and phospho-mTOR (Ser2448) in a dose-dependent manner.

In this study, we report that RM-3-22 inhibits the PI3K/Akt/mTOR pathway, inducing autophagy, which was confirmed by the identification of lipidation of MAP1LC3B and inhibition of SQSTM1 in a dose-dependent way. Our *in vivo* study also revealed the increased lipidation of MAP1LC3B. Furthermore, combinatorial treatments of RM-3-22 with autophagy inhibitor 3-MA reversed the fatal effect caused by RM-3-22 in A549 cells, while the autophagy inducer rapamycin stimulated the autophagy flux and reduced cell viability in A549 cells. These findings suggest that RM-3-22 induces autophagy-mediated cell death in A549 cells. Furthermore, the application of an inducer of PI3K/Akt/mTOR negatively impacted the occurrence of autophagy. This observation further suggested that the induction of autophagy by the compound is mediated by the PI3K/Akt/mTOR pathway.

Moreover, target apoptosis is still one of the key mechanisms to inhibit cancer progression. This is mediated by the disruption of the loss of mitochondrial membrane potential and the nuclear translocation of Bax, resulting in the release of cytochrome C into the cytosol and triggering the apoptotic cascade (Chaudhry et al., 2022). The treatment of RM-3-22 stimulated the cleavage of caspase 3, caspase 7, caspase 9, and PARP-1, which is symbolic of apoptotic death. Auxiliary observations included the downregulation of Bcl2, the translocation of BAX from the cytosol to the mitochondria, and comprehensive caspase-dependent apoptotic cell death in A549 cells. The cleavage of PARP-1 was also observed *in vivo*.

Multiple research studies explained that cyclin B1 and CDC2 downregulation leads to G2/M phase arrest of the cell cycle. Mechanistically, the induction of p21 directly inhibits the function of CDC2, destroying CDC2 and cyclin B1 (F Bunz et al., 1998). In addition, the reduction in p-CDC25C (ser216) leads to the inability of the CDC25 phosphatase to dephosphorylate and activate CDC2, resulting in G2/M arrest (Liu et al., 2020; Zhang et al., 2021; Peng et al., 2016). Our compound, RM-3-22, also prompted G2/M arrest in the A549 cell line by the destruction of cyclin B1, CDC2, and p-CDC25C (ser216) and the upregulation of p21. Moreover, the downregulation of cyclin B1 was observed *in vivo*. Furthermore, our study aimed to elucidate the interactions among these pathways in the regulation of tumor progression in NSCLC.

Furthermore, we have tried to find the supreme pathway that regulates other pathways. Time-dependent RM-3-22 exposure underlines the primacy of autophagy, followed by its exposure. We employed the autophagy inhibitor 3-MA and the autophagy activator rapamycin to further check the role of autophagy in the regulation of other pathways. We have noticed that the application of the autophagy inhibitor reversed the cyclin B1 reduction caused by RM-3-22. In addition, the cleavage of PARP-1 is reversed by 3-MA. These observations infer the supremacy of autophagy over other pathways.

Again, RM-3-22 elicits the upregulation of FTH1. Importantly, FTH1 upregulation appears to play a controversial role in different cancers (Pham et al., 2004; Liu et al., 2011; Faniello et al., 2008). However, in NSCLC, specifically in A549 cells, it is reported to act as a tumor suppressor gene and exerts its suppressive role by inducing miR-125b/p53-mediated apoptosis (Biamonte et al., 2018). Notably, from the KM plotter, we found that its downregulation negatively affects the survival of lung carcinoma, and its expression also seems to be slightly downregulated during lung cancer progression (Luo et al., 2024). Thereby, based on the publicly available data and previous reports, we have considered FTH1 a tumor suppressor in lung cancer, and our molecule is potent enough to induce its expression. We also observed that FTH1 knockdown leads to an increase in the aggressiveness of A549 cells in this context. However, more investigation is needed to explore how FTH1 exerts its tumor-suppressive role at the molecular level in the current context. This novel observation might open a new therapeutic strategy to treat cancer.

With a distinct mode of action and superior anticancer properties, RM-3-22 has a great deal of promise for use in clinical settings in the future. It might be investigated as a targeted treatment, either alone or in conjunction with current treatment plans, for aggressive and drug-resistant malignancies. Its capacity to trigger autophagy also raises the possibility of using it to overcome therapeutic resistance, which is a significant obstacle in the treatment of cancer. The clinical translation of RM-3-22 as a next-generation HDAC inhibitor may be facilitated by future research concentrating on *in vivo* validation, pharmacokinetics, and combinatorial approaches. To guarantee its clinical viability, possible off-target effects and long-term safety must also be evaluated.

## 5 Conclusion and future perspectives

**FIGURE 8:**
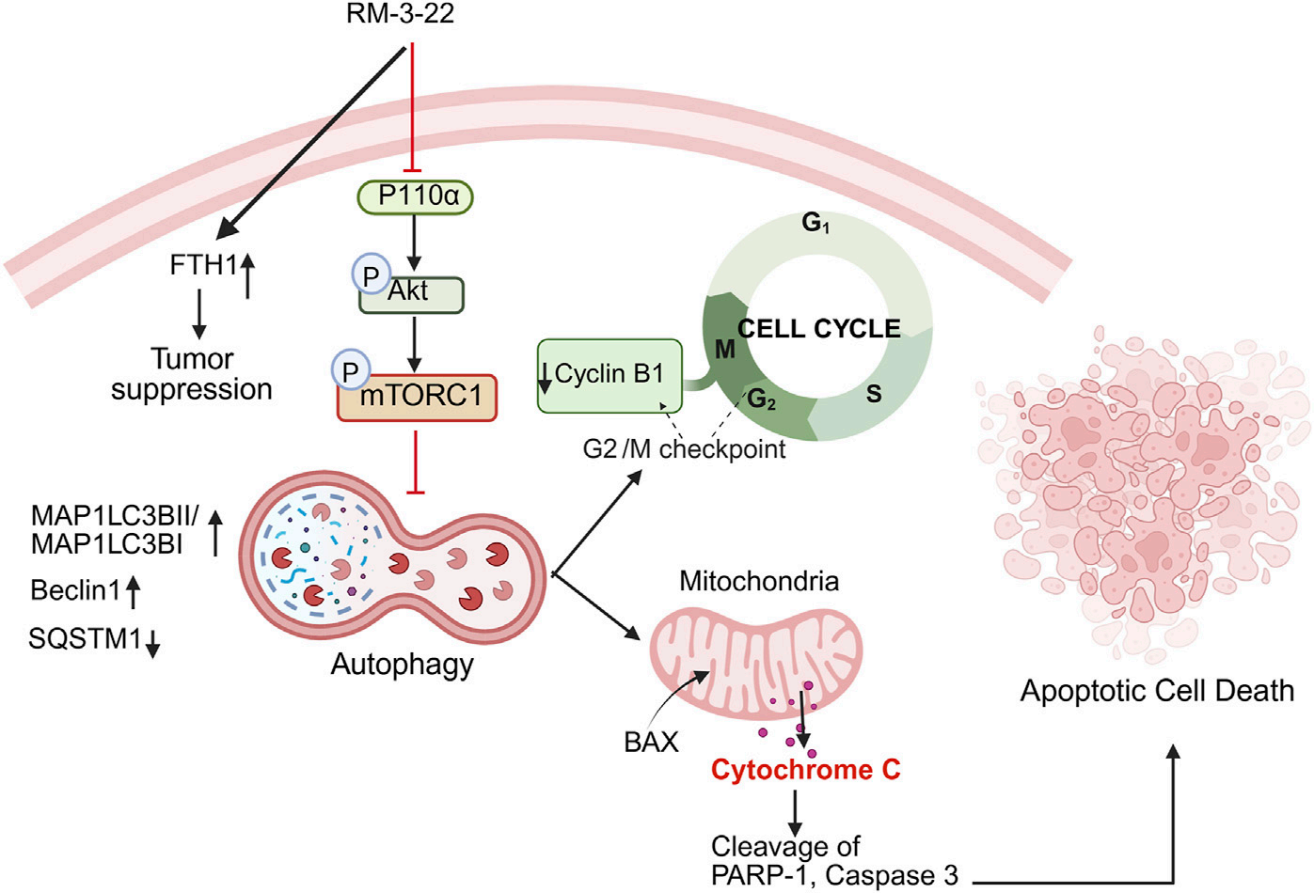
Summary of the mechanism of action after RM-3-22 treatment. The diagram indicates that RM-3-22 is responsible for the inhibition of the PI3K/Akt/mTOR pathway, which leads to the activation of the autophagy pathway, marked by the induction of regulatory makers of autophagy, MAP1LC3B II and Beclin1, and also the degradation of p62. The induction of the autophagy pathway further induces the apoptosis pathway, confirmed by the translocation of BAX from the cytosol to the nucleus, and subsequently the cleavage of PARP-1 and caspase 3. RM-3-22-induced autophagy is further responsible for the G2/M cellcycle arrest, and this is confirmed by the destruction of cyclin B1 and CDC2 in a dose-dependent way. Apart from that, RM-3-22 activates FTH1-related tumor suppression in NSCLC.

Our research elaborated on the interconnection between the PI3K/Akt/mTOR pathway, autophagy, cell cycle arrest, and apoptosis in NSCLC. Additionally, we affirmed FTH1 as a tumor suppressor, and it could be a promising target for lung cancer. We explored the PI3K/Akt/mTOR-dependent autophagy pathway that leads to G2/M arrest and activation of caspase-dependent apoptosis (Figure 8). Most importantly, in our quest to discover a novel therapeutic agent with great potential to treat lung cancer, RM-3-22 emerges as a promising therapeutic agent for treating lung cancer.

For further enhancement of RM-3-22 toward clinical applications, the conduction of comprehensive studies, including pharmacokinetics and pharmacodynamics (PK/PD) assessments, toxicity evaluations in larger models, and investigations in combination therapies, are required. These actions will pave the way for future clinical trials and emphasize the translational potential of RM-3-22 in the lung cancer domain.

## Data Availability

The original contributions presented in the study are included in the article/supplementary article. Further inquiries can be directed to the corresponding author.

## Supplementary material

SUPPLEMENTARY FIGURE 1 Synthetic route of RM-3-22. Reagents and conditions: **(a)** i) SOCl_2_, toluene, reflux, rt; ii) epsilon-caprolactam, toluene, rt; **(b)** Fe, NH_4_Cl, ethanol: water: 9: 1, reflux; **(c)** (E)-methyl 3-(4-(bromomethyl) phenyl) acrylate, K_2_CO_3_, DMF, 60°C; **(d)** 1M LiOH(aq), p-dioxane, rt; **(e)** NH_2_OTHP, EDC.HCl, HOBt, DIPEA, DMF, rt; **(f)** 10% TFA (aq), MeOH, rt.

SUPPLEMENTARY FIGURE 2 RM-3-22 induces autophagy. **(A)** AO staining was carried out in A549 cells through exposure to different concentrations of RM-3-22 along with SAHA for 24 h. **(B)** Expression of autophagy pathway-related genes, Beclin1, ATG5, ATG7, and RB1CC1 in the mRNA level was assessed after exposure to RM-3-22 at different concentrations along with SAHA at a 500 nM dose, which was carried out by RT-PCR. Data are presented as the mean ± SD of three independent experiments; *p < 0.05, **p < 0.01, and ***p < 0.001 compared with untreated control. **(C)** mRNA level expression of ATG7 and Beclin1 increased in the spheroid configuration of A549 cells upon treatment with RM-3-22 in a time-dependent manner. Data represent the mean  ±  SD on three independent experiments (one-way ANOVA, *p < 0.05, **p  <  0.01, ***p  <  0.001). **(D)** Different concentrations of rapamycin and 3-MA were administered to A549 cells to detect the safe dose for further study. **(E)** Bright-field imaging of A549 cells in 2D and 3D forms treated with or without RM-3-22, rapamycin, and 3-MA for 24 h. Bright-field imaging showed that the reduction in cell viability by RM-3-22 was potentiated by rapamycin and reduced by 3-MA. The efficiency of the size reduction in spheroid by RM-3-22 was also induced by rapamycin treatment and reduced by 3-MA treatment. **(F)** Application of autophagy inducer rapamycin with RM-3-22 synergistically decreases the A549 cells survival (I), whereas autophagy inhibitor 3-MA recovers the cell mortality caused by RM-3-22. Data represent the mean  ±  SD on three independent experiments (one-way ANOVA, *p < 0.05, **p  <  0.01, ***p  <  0.001).

SUPPLEMENTARY FIGURE 3 RM-3-22 induces apoptosis. **(A)** Flow cytometric analysis using rhodamine 123 dye shows a decrease in the MMP level after exposure to RM-3-22 in a concentration-dependent manner. Data represent the mean  ±  SD on three independent experiments (one-way ANOVA, *p < 0.05, **p  <  0.01, ***p  <  0.001). **(B)** DNA fragmentation assay shows an increase in fragmented DNA in higher concentrations of RM-3-22. **(C)** Bcl2 decreased in A549 cells with an increase in the concentration of RM-3-22. Data are presented as the mean ± SD of three independent experiments; one-way ANOVA *p < 0.05, **p < 0.01, ***p < 0.001, compared with untreated control.

## Abbreviations

HDACi, histone deacetylase inhibitor; NSCLC, non-small-cell carcinoma; SAHA, suberoylanilide hydroxamic acid; MTT, 3-(4, 5-dimethyl thiazolyl-2)-2, 5-diphenyltetrazolium bromide; PI3K, phosphoinositide-3-kinase; mTOR, mammalian (or mechanistic) target of rapamycin; SQSTM1, sequestosome-1; ATG, autophagy-related gene; LC3B, microtubule-associated protein 1 light chain 3 beta; 3-MA, 3-methyl adenine; Bcl2, B-cell lymphoma 2; BAX, Bcl-2-associated X protein; caspase, cysteine-aspartic acid proteases; PARP, poly(ADP-ribose) polymerases; CYT C, cytochrome C; FTH1, ferritin heavy chain 1; CDC2, cell division control 2.
