# Supplementary figures and images for: Anticancer activity of RM-3-22: a TAZQ-based hydroxamic acid derivative targeting NSCLC in vitro and in vivo
Source: Front Pharmacol. 2025 Jun 5;16:1544666. doi: 10.3389/fphar.2025.1544666 (PMC12176848; doi:10.3389/fphar.2025.1544666)

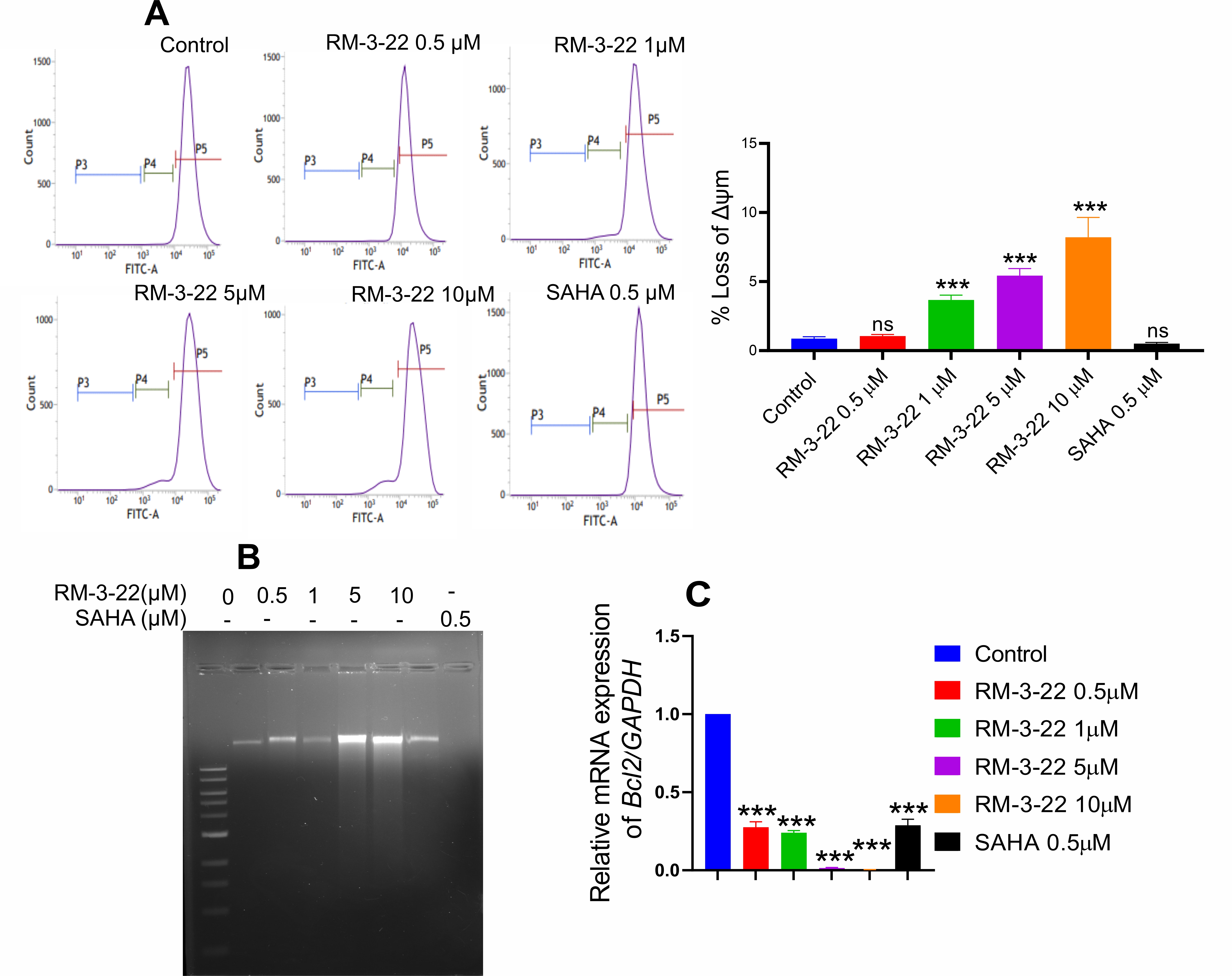

Supplement: Supplementary file 1 [file Image3.jpeg]

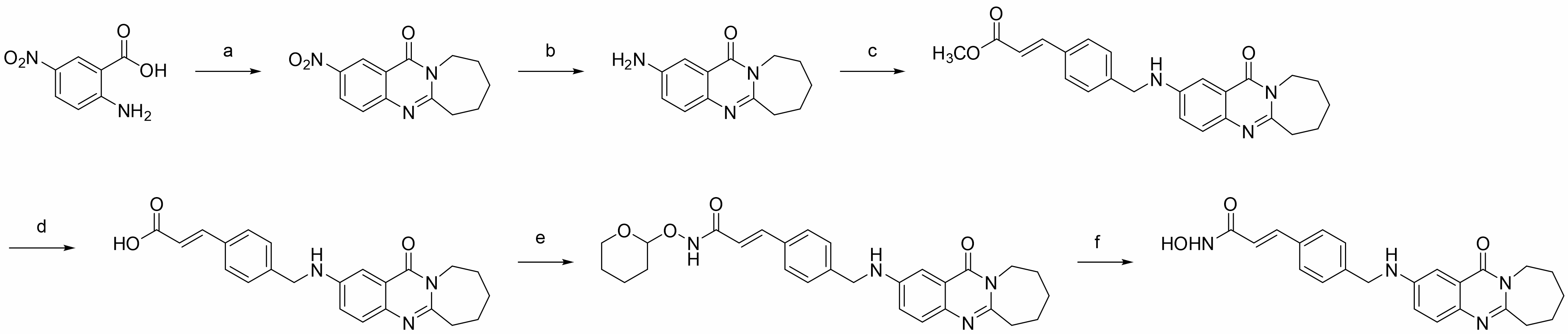

Supplement: Supplementary file 3 [file Image1.jpeg]

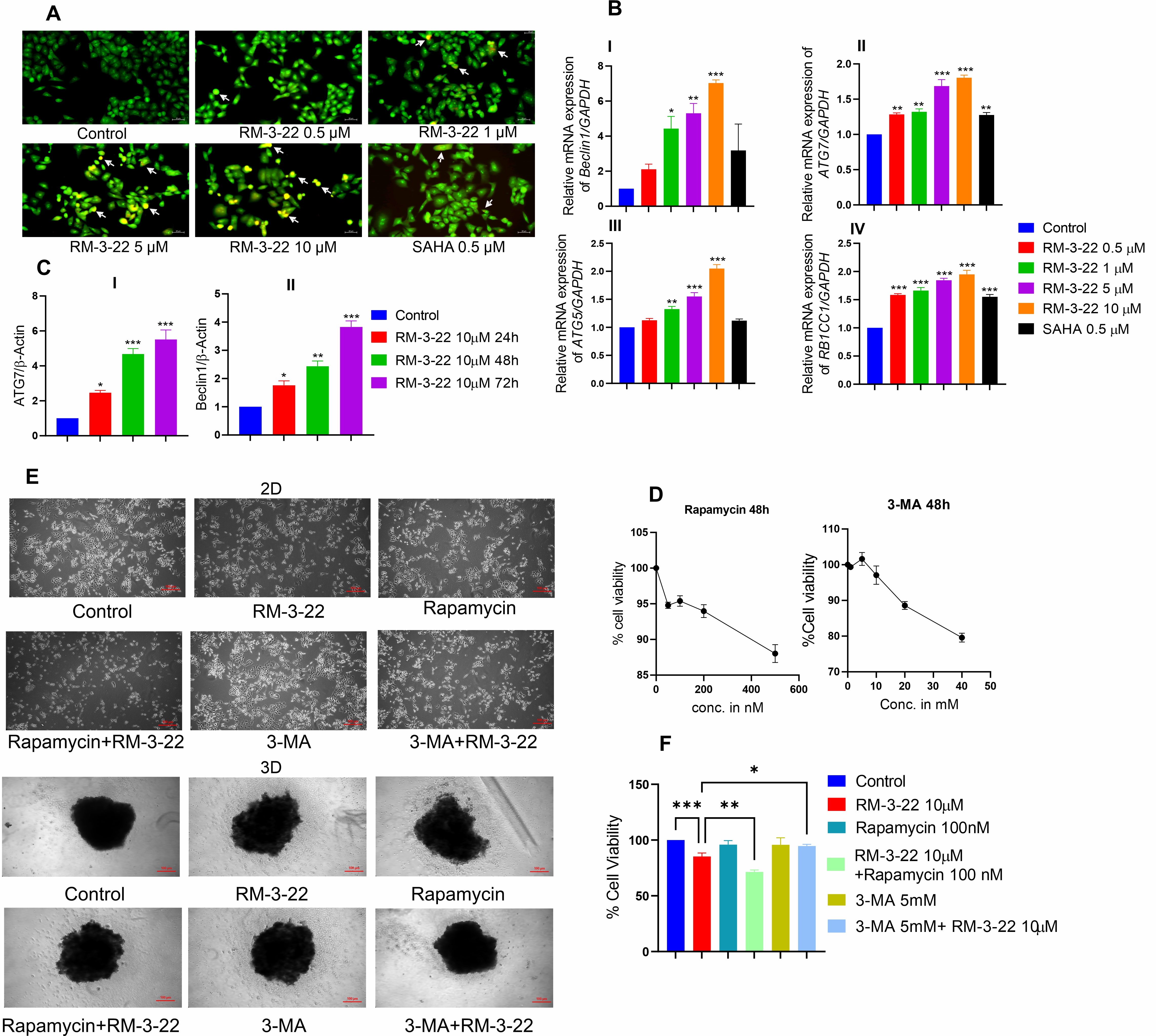

Supplement: Supplementary file 4 [file Image2.jpeg]
