# Supplementary material for: Anticancer activity of RM-3-22: a TAZQ-based hydroxamic acid derivative targeting NSCLC in vitro and in vivo
Source: Front Pharmacol. 2025 Jun 5;16:1544666. doi: 10.3389/fphar.2025.1544666 (PMC12176848; doi:10.3389/fphar.2025.1544666)
Supplement: Supplementary file 2 [file Table1.docx]

**Supplementary Table 1:**

**Table S1: List of Antibodies of the work done**

| **Name of the Antibody** | **Manufacturer** | **Cat. no** |
| --- | --- | --- |
| P-mTOR (ser 2448) | Cell Signaling Technology | 5536 |
| Total mTOR | Cell Signalling Technology | 2983 |
| P-AKT (ser473) | Cell Signaling Technology | 4060 |
| Total AKT | Cell Signaling Technology | 9272 |
| PI3 Kinase p110α | Cell Signalling Technolong | 4249 |
| Beclin1 | Cell Signaling Technology | 3495 |
| SQSTM1 | Cell Signaling Technology | 39749 |
| MAP1LC3B | Cell Signaling Technology | 4108 |
| P-4EBP1 | Cell Signaling Technology | 2855 |
| CYCLIN B1 | Cell Signaling Technology | 4138 |
| p-CDC25C (Ser216) | Cell Signaling Technology | 4901 |
| CDC2 | Cell Signaling Technology | 28439 |
| P21 | Cell Signaling Technology | 2947 |
| BCL2 | Santa Cruz Biotechnology | Sc-7382 |
| BID | Santa Cruz Biotechnology | Sc-373939 |
| CASPASE 9 | Santa Cruz Biotechnology | Sc-17784 |
| CASPASE 7 | Cell Signaling Technology | 12827 |
| CASPASE 3 | Cell Signaling Technology | 14220 |
| PARP1 | Cell Signaling Technology | 9542 |
| PARP-1 | Santa Cruz Biotechnology | Sc-8007 |
| CYTOCHROME C | Santa Cruz Biotechnology | sc-13156 |
| BAX | Santa Cruz Biotechnology | Sc-7480 |
| COXIV | Cell Signaling Technology | 4850 |
| FTH1 | Cell Signaling Technology | 4393 |
| CASPASE 3 | Santa Cruz Biotechnology | Sc-7272 |
| β-Actin | Novus Biologicals | NB600532H |
| SECONDARY ANTIBODY RABBIT | Cell Signaling Technology | 7074 |
| SECONDARY ANTIBODY MOUSE | Cell Signaling Technology | 7076 |

**Supplementary Table: 2**

**Table S2: List of Primers used in the work done**

| **Gene** | **Primer** | |
| --- | --- | --- |
|  | **Forward** | **Reverse** |
| BECLIN1 | 5'-GCGACTGTGATGCGCTAAT-3' | 5'-TAGGGTTCCTCTTGGAGAA-3' |
| ATG5 | AAAGATGTGCTTCGAGATGTGT | CACTTTGTCAGTTACCAACGTCA |
| ATG7 | ATGATCCCTGTAACTTAGCCCA | CACGGAAGCAAACAACTTCAAC |
| RB1CC1 | 5’-GAAAGAGCTTGCTCAGGGATT-3’ | 5’-TCATCAACTGATTTGCGTGACT-3’ |
| FTH1 | 5’-CCCCCATTTGTGTGACTTCAT-3’ | 5’GCCCGAGGCTTAGCTTTCATT-3’ |
| BCL2 | 5′-CTACCTCCACCATGCCAAGT-3′ | 5′-GCAGTAGCTGCGCTGATAGA-3′. |
